# Trimester-specific reference intervals for thyroid function parameters in pregnant Caucasian women using Roche platforms: a prospective study

**DOI:** 10.1007/s40618-023-02098-0

**Published:** 2023-04-24

**Authors:** R. M. Dorizzi, G. Spiazzi, N. Rolli, P. Maltoni, L. Mingolla, C. Sgarzani, M. Torello, F. Tosi, C. Bonin, P. Moghetti

**Affiliations:** 1 Clinical Pathology Unit, Hub Laboratory, AUSL Romagna, Cesena, Italy; 2 Unit of Endocrinology, Diabetes and Metabolism, University of Verona and Azienda Ospedaliera Universitaria Integrata Verona, p.le Stefani 1, 37126 Verona, Italy; 3 Obstetrics and Gynecology, Azienda Ospedaliera Universitaria Integrata Verona, Verona, Italy

**Keywords:** Thyroid function, TSH, FT4, FT3, Pregnancy, Reference interval

## Abstract

**Background:**

Standard thyroid function parameters reference intervals (RI) are unsuitable during pregnancy, potentially resulting in incongruous treatments that may cause adverse effects on pregnancy outcomes. We aimed at defining trimester-specific TSH, FT4 and FT3 RI, using samples longitudinally collected from healthy Caucasian women.

**Materials and methods:**

Blood samples from 150 healthy Caucasian women, who had a physiological gestation and a healthy newborn at term, were collected in each trimester and at around six months post-partum. They showed mild iodine deficiency. After excluding women with overt TSH abnormalities (> 10 mU/L) and/or TPO antibodies, data from 139 pregnant women were analyzed by means of widely used Roche platforms, and TSH, FT4 and FT3 trimester-specific RI were calculated. Post-partum data were available for 55 subjects.

**Results:**

Serum TSH RI were 0.34–3.81 mU/L in the first trimester, and changed slightly to 0.68–4.07 U/L and 0.63–4.00 mU/L in the second and third trimester, respectively. Conversely, both FT4 and FT3 concentrations progressively decreased during pregnancy, the median values in the third trimester being 14.8% and 13.2% lower, respectively, than in the first trimester. Thyroid function parameters in the first trimester were similar to those measured after the end of pregnancy.

**Conclusions:**

This study calculates trimester-specific RI for thyroid function parameters in pregnancy, and proposes the reference limits that should be adopted when using Roche platforms in Caucasian women.

**Supplementary Information:**

The online version contains supplementary material available at 10.1007/s40618-023-02098-0.

## Introduction

Thyroid hormones (TH) are essential for physiological pregnancy and fetal development. Importantly, since the fetal thyroid gland is not functionally mature until week 20 of gestation, the fetus is dependent on the placental transfer of thyroxine [1]. The supply of TH to the fetus is influenced by the increase of thyroxine-binding globulin (TBG) concentration that occurs in pregnancy due to higher estrogen concentrations, and by the degradation of TH by placental type 3 iodothyronine deiodinase. Furthermore, a higher urinary iodine excretion, due to increased glomerular filtration rate, also occurs [2]. Early in the first trimester of pregnancy, placental production of human chorionic gonadotropin (hCG), a weak thyroid stimulating hormone (TSH) agonist, stimulates the release of TH, which in turn may lower TSH [3]. During gestation, as a consequence of physiological changes, TH production and daily iodine requirements increase by about 50% [3–5].

These pregnancy-specific changes, along with the increased demand for TH, often reveal pre-existing mild thyroid dysfunction, which appears as gestational thyroid disease. While overt maternal hypothyroidism occurs in 0.2–0.6% of pregnant women, maternal subclinical hypothyroidism may be found in 3.5% to ~ 18% of all pregnancies [1]. Since these conditions may recur in the postpartum period, surveillance of these subjects is appropriate also after the end of pregnancy [6].

A higher risk of pregnancy complications, including preterm delivery, low birthweight, miscarriage and pre-eclampsia, and potential detrimental effects on fetal neurodevelopment are associated with overt and possibly subclinical maternal thyroid dysfunction [1, 7]. Therefore, recognizing these alterations in pregnant women is a very important issue. Unfortunately, the physiological changes in thyroid metabolism during pregnancy, coupled with differences between available measurement methods and other factors, such as the genetic characteristics of populations and the relative iodine intake of subjects, make the thyroid function parameters (TFP) reference intervals used in non-pregnant subjects unsuitable for pregnant women.

According to the current ATA 2017 guidelines, a TSH upper reference limit of 4.0 mU/L, approximately reduced by 0.5 mU/L as compared to the non-pregnant limit, should generally be applied in early pregnancy, with a gradual return towards the non-pregnant limit in the second and third trimesters, independent of assay methods used [8]. Accordingly, many laboratories now rely on these fixed reference intervals. However, available evidence suggests some differences may exist, and should ideally be taken into account when interpreting the results. Moreover, little information is available on trimester-specific FT4 and FT3 intervals, which may sometimes be crucial in appropriately managing thyroid dysfunction in pregnancy, especially in the presence of undetectable TSH levels.

The present longitudinal study was conducted to further improve information on TFP reference intervals during pregnancy, by analyzing healthy women with a physiological pregnancy and birth at term of a healthy baby, using the Roche reagents and instrumentation, which are used worldwide.

The importance of this study is manifold, since it provides information regarding: (a) the reference intervals specific to the different trimesters of pregnancy of all clinically important TFP; (b) the physiological changes during the pregnancy of these parameters in the absence of any therapeutic interventions. Notably, to our knowledge this study represents the first longitudinal study carried out in strict accordance with the current EP28-A3c CLSI standard [9].

## Materials and methods

The subjects included in this analysis were enrolled in the Trilogy Study, a prospective study in pregnancy conducted in Verona, north-east Italy, on a sample of over 500 nondiabetic women, the vast majority of whom of Caucasian ethnicity. The primary objective of the project was to identify predictive factors for gestational diabetes and pre-eclampsia in a non-selected cohort of women.

For the purpose of the present ancillary study, 150 women from the Trilogy Study cohort were selected, all characterized by Caucasian ethnicity, no personal history of thyroid diseases or other major diseases, a physiological course of pregnancy and full-term birth of a single healthy and normal weight baby.

The women recruited in the study underwent prospective clinical evaluations and the collection of blood at three different times during pregnancy: 14–16, 24–26 and 30–32 weeks, to represent, respectively, trimester 1 (T1), 2 (T2), and 3 (T3) of pregnancy. A urine sample was also collected at T1 and an additional blood sample was scheduled between 20 and 30 weeks postpartum, to represent the non-pregnant state. During the visits scheduled at each time point of the protocol, information was systematically collected on any pathologies occurring during pregnancy and, after the end of pregnancy, on the timing and modalities of delivery, on maternal complications and on the characteristics of the newborn. In women who missed the post-pregnancy visit, these data were collected by consultation of the CedAP certificate, a national electronic register of the Italian Ministry of Health which records information on all pregnancies, including maternal diseases and complications, delivery modalities, newborn birthweight and clinical status.

Serum TSH, FT4 and FT3 were measured in these women in the three trimesters of gestation and in the post-partum samples. In addition, Anti-Thyroid Peroxidase antibodies (TPO) and spot urinary iodine were also measured in the first trimester, to assess the thyroid autoimmunity status and the iodine status of the study population, respectively.

In analyzing the data for the purpose of defining the reference intervals, the subjects who had values of TSH > 10 mU/L (n = 1) or TPO positivity (n = 10) were excluded; the final analyses were therefore conducted on 139 subjects. The post-pregnancy sample was obtained in 55 of these women, as many subjects missed the post-pregnancy visit.

The Human Research Ethics Committee at the Hospital Trust of Verona approved the study, and written informed consent was obtained from each study participant.

All serum and urine samples were immediately frozen at − 80 °C after collection until analysis and TSH, FT4, FT3, TPO and urinary iodine were assayed as a batch.

Serum TSH, FT4, FT3 and TPO were measured by an electrochemiluminescence analyser, Modular Analytics E170, and Elecsys Cobas Reagents (Roche Diagnostics, Milan, Italy).

Urine iodine concentration was measured by the Inductively Coupled Plasma Mass Spectrometry technology using the X Series 2 ICP-MS (Thermo Fisher Scientific, Waltham, Mass, USA). The status of iodine adequacy/deficiency in this cohort was established according to WHO criteria [5].

### Comparison of results with other available prospective studies of thyroid function in pregnancy

While numerous cross-sectional studies were carried out between 2010 and 2021 in samples randomly taken in each trimester (referenced in Supplemental Material 1), the number of longitudinal studies of thyroid function in pregnancy, performed following strict criteria of selection, is scarce.

For a comparison of our findings with previous longitudinal studies carried out in pregnancy, we searched MEDLINE using various combinations of the following search terms: ‘thyroid function’, ‘FT4’, ‘thyroxine’, ‘TSH’, ‘thyrotropin’, ‘pregnancy’, ‘gestation’, ‘reference range’ and ‘reference interval’, for the articles published between January 2010 and December 2021. Studies published before 2010 were not considered since analyzers/reagents employed were no longer available or substantial methodological changes had been made. We also sourced additional publications from references in individual articles. The studies were selected if they: were in English; investigated pregnant women longitudinally; measured TFP using one of four widely available assay methods: Abbott Architect, Beckman Dxl, Roche Elecsys and Siemens Advia Centaur; reported reference intervals as 2.5–97.5 percentiles; measured Anti-TPO and/or Anti-Tg antibodies, and these were negative. Data from the selected papers were summarized in Tables, reporting first author, year of publication, method, age distribution of the subjects, ethnicity, country of study, information on the assessment of relevant clinical features (thyroid ultrasonographic characteristics, anti-thyroid antibodies, and iodine deficiency), the number of subjects examined in the three trimesters of pregnancy, and TSH, FT4 and FT3, when available, reference intervals in the different trimesters of pregnancy and in the post-partum, when available.

### Statistical analysis

Data were analyzed using Medcalc© software (Ostende, Belgium). Results of parameters were expressed as median (M), 2.5th percentile (P2.5) and 97.5th percentile (P97.5). The limits of the reference intervals were calculated by three methods: parametric, non-parametric (P2.5–P97.5) and “robust”, using the statistical module of the software following the manufacturer’s instructions. The data were tested for normality using the Kolmogorov Smirnov test.

The distribution of TSH concentration was not normal in the three trimesters, while the distribution of FT4 and FT3 was normal.

The calculation of the trimester-specific reference concentrations in pregnancy was carried out following the recommendations of the Clinical and Laboratory Standards standard EP28-A3c [9]. CLSI standard recommends a minimum sample size of 120 reference subjects that allows 90% confidence limits to be computed non-parametrically, suggesting as an alternative the “robust method”, proposed by Horn and Pesce [10, 11], when sample size is lower than 120 units and when the analytical data do not follow a Gaussian distribution.

The Horn and Pesce robust method is based on the transformation of the original data according to Box and Cox, followed by a “robust” algorithm giving different weights to the data, depending upon their distance from the mean. This method allows for the calculation of the reference limits from a limited number of observations providing 90% Confidence Intervals (CIs) around the limits, using “the bootstrap method” which is a “resampling” method that creates a “pseudosample” from the data [11, 12]. Therefore, the reference intervals of the three pregnancy trimesters were calculated using the non-parametric method, and that of the smaller (n = 55) post-pregnancy sample using the robust method. The results obtained by using the robust method were compared with those obtained with the parametric and the non-parametric method. Further details regarding the methods employed are provided in the Supplemental Material 2.

A comparison between the different trimesters was carried out with the Wilcoxon test for paired samples.

## Results

In this cohort, the mean and median iodine values were 79.7 and 79 ug/L, respectively, consistent with a condition of mild iodine deficiency.

The trimester-specific mean, median and coefficient of skewness and kurtosis of serum TSH, FT4 and FT3 concentrations for healthy pregnant women, with negative TPO, are shown in Tables A–C, Supplemental Material 3.

The reference intervals of TFP by the different statistical methods, carried out as recommended by CLSI EP28-A3c, are detailed in Tables 1, 2 and 3. The results obtained by using these different statistical methods were very similar, and the small observed differences may be considered clinically trivial.

**Table 1:** Reference interval of TSH (mU/L)

	First trimester	Second trimester	Third trimester	Postpartum
Sample size	139	139	139	55
Mean + 2SD				
Lower limit	0.37	0.70	0.65	0.58
90% CI	0.26–0.49	0.60–0.80	0.54–0.76	0.47–0.71
Upper limit	3.91	3.97	3.91	3.32
90% CI	3.61–4.22	3.65–4.31	3.61–4.21	2.87–3.84
Percentile non parametric method				
Lower limit	0.34	0.68	0.63	0.53
90% CI	0.07–0.55	0.58–0.85	0.32–0.83	–*
Upper limit	3.81	4.07	4.00	3.33
90% CI	3.62–4.81	3.45–4.69	3.51–4.37	–*
Robust method				
Lower limit	0.35	0.687	0.631	0.561
90% CI	0.24–0.48	0.560–0.79	0.54–0.74	0.46–0.68
Upper limit	3.95	4.02	3.94	3.42
90% CI	3.63–4.25	3.71–4.33	3.62–4.24	2.92–3.90

–*Not calculated, as recommended by CLSI document EP28-A3c [16]

**Table 2:** Reference interval of FT4 (pmol/L)

	First trimester	Second trimester	Third trimester	Post-partum
Sample size	139	139	139	55
Mean + 2SD				
Lower limit	11.39	9.98	9.48	11.97
90% CI	11.09–11.70	9.71–10.24	9.19–9.80	11.53–12.43
Upper limit	17.39	15.25	15.10	19.10
90% CI	16.96–17.84	14.86–15.65	14.71–15.49	18.06–20.26
Percentile non parametric method				
Lower limit	11.35	9.79	9.48	11.95
90% CI	10.08–12.14	9.24–10.3	8.20–10.12	–*
Upper limit	17.35	15.47	15.37	19.25
90% CI	16.80–19.05	14.88–16.90	14.43–16.25	–*
Robust method				
Lower limit	11.35	9.96	9.46	11.88
90% CI	11.06–11.67	9.68–10.25	9.16–9.77	11.50–12.33
Upper limit	17.43	15.30	15.13	19.29
90% CI	16.96–17.89	14.90–15.70	14.73–15.54	18.22–20.38

–*Not calculated, as recommended by CLSI document EP28-A3c [16]

**Table 3:** Reference interval of FT3 (pmol/L)

	First trimester	Second trimester	Third trimester	Post-partum
Sample size	139	139	139	55
Mean + 2SD				
Lower limit	3.88	3.61	3.59	4.18
90% CI	3.73–4.02	3.53–3.71	3.51–3.67	4.04–4.33
Upper limit	6.05	5.38	5.37	6.33
90% CI	5.92–6.17	5.25–5.50	5.23–5.52	6.04–6.66
Percentile non parametric method				
Lower limit	3.74	3.53	3.49	3.97
90% CI	3.46–4.09	3.32–3.72	3.36–3.75	–*
Upper limit	6.06	5.54	5.48	6.71
90% CI	5.84–6.64	5.13–5.74	5.22–5.71	–*
Robust method				
Lower limit	3.87	3.61	3.58	4.16
90% CI	3.71–4.0	3.51–3.70	3.50–3.66	4.00–4.34
Upper limit	6.06	5.39	5.39	6.41
90% CI	5.94–6.20	5.26–5.52	5.23–5.54	6.06–6.76

–*Not calculated, as recommended by CLSI document EP28-A3c [16]

The TSH reference interval (2.5th–97.5th percentiles) of our population was 0.34–3.81 mU/L in the first trimester of pregnancy, 0.68–4.07 mU/L in the second trimester and 0.63–4.00 mU/L in the third trimester. In 55 of these subjects, re-examined about six months after delivery, the TSH reference interval (robust method) was 0.56–3.42 mU/L.

The FT4 reference interval in this cohort was 11.35–17.35 pmol/L (8.82–13.48 ng/L) in the first trimester of pregnancy, 9.79–15.47 pmol/L (7.60–12.02 ng/L) in the second trimester and 9.48–15.37 pmol/L (7.37–11.94 ng/L) in the third trimester. In subjects re-examined six months after delivery, the FT4 reference interval (robust method) was 11.88–19.29 pmol/L (9.29–14.96 ng/L).

Finally, the FT3 reference interval (2.5th–97.5th percentiles) was 3.74–6.06 pmol/L (2.43–3.94 ng/L) in the first trimester of pregnancy, 3.53–5.54 pmol/L (2.30–3.61 ng/L) in the second trimester and 3.49–5.48 pmol/L (2.27–3.57 ng/L) in the third trimester. In subjects re-examined six months after delivery, the FT3 reference interval (robust method) was 4.16–6.41 pmol/L (2.71–4.17 ng/L).

**Fig. 1:**
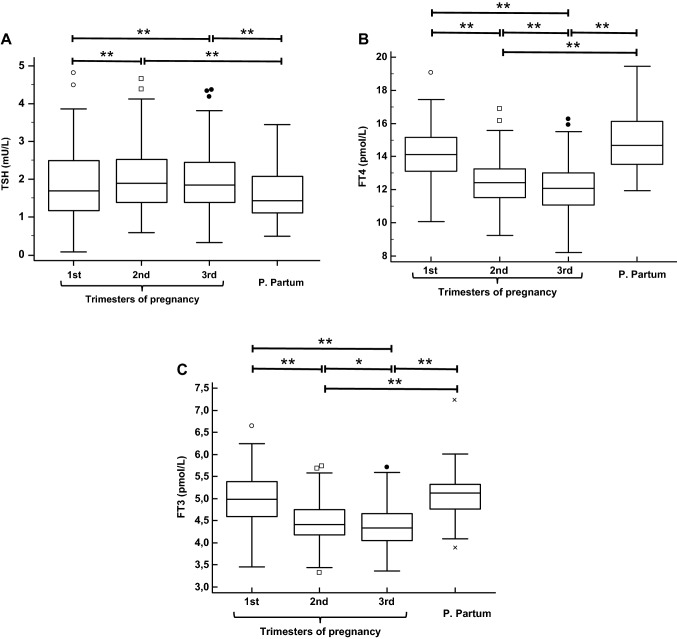
Box and whisker plots of the TSH (**A**), FT4 (**B**) and FT3 (**C**) serum concentrations in the three trimesters of pregnancy and in the post-partum. In these plots, the horizontal lines inside each box represent the medians, while the box frames include the interquartile ranges (25–75 percentile). The vertical lines extend from the minimum to the maximum values excluding outside values, defined as values smaller than the lower quartile minus 1.5 times the interquartile range, or greater than the upper quartile plus 1.5 times the interquartile range, which are displayed as separate points. The paired results of each time point were compared by the Wilcoxon test. *P < 0.05–0.011; **P ≤ 0.010–0.001

Figure 1 compares, as box and whisker plots, the TSH, FT4 and FT3 concentrations in the three trimesters of pregnancy and in the post-partum period. In paired comparisons, a significant increase of TSH concentration and a significant decrease of FT4 and FT3 concentration were found during the pregnancy. However, there were no significant differences between the (late) first trimester and the corresponding post-pregnancy TSH, FT4 and FT3 concentrations.

During pregnancy, TSH concentration in T1 was slightly but significantly lower than in T2 and T3, while a statistically significant difference was not found between T2 and T3. Conversely, FT4 and FT3 concentrations in T1 were significantly higher compared to T2, and concentrations in T2 were significantly higher compared to T3.

Table 4 summarizes the characteristics of the eight longitudinal studies published before the present paper, between January 2010 and December 2021, which calculated the reference intervals of thyroid function in pregnancy: five employed Roche, three employed Abbott, two employed Beckman, and one employed Siemens instrumentation [13–20]. Three of them (two using Roche and one Abbott) did not include FT3 results. Table 5 compares the results of the present study with those of previous longitudinal studies.

**Table 4:** Characteristics of the present and previous longitudinal studies on the reference intervals for thyroid function in pregnancy

Authors, year (Refs.)	Method	Age ± SD (range)	Ethnicity	Country	Ecography/Ab	Iodine assay	Subjects	Week 1 trim	Week 2 trim	Week 3 trim
**Present study**	**Roche Elecsys**	33.7 ± 3.8 (23–42)	Caucasian	Italy	No/TPO	Yes (ICPM°)	139	**14–16**	**24–26**	**30–32**
Sekhri et al. 2016 [13]	Roche Elecsys	24.5 ± 3.5 (18–45)	Indian	India	No/TPO	Yes (SK reaction°°)	86	10.8(± 2.4)	NR	NR
Joosen et al. 2016 [14]	Roche Elecsys	Not reported	Caucasian	Netherlands	No/TPO	No	97	9–13	27–29	36–39
Ortega Carpio et al. 2018 [15]	Roche Elecsys	30.7	Caucasian	Spain	No/TPO	No	145, 105, 67*	9–11	26–28	34–36
Kostecka-Matyja et al. 2017 [16]	Roche Elecsys	35 (27–47)	Caucasian	Poland	No/TPO	No	172	NR	NR	NR
Yuen et al. 2020 [17]	Roche Elecsys	31.1 ± 3.9	Chinese	Hong Kong	No/TPO	Yes (ICPM°)	NR**	12–13	16–20	32–36
Yuen et al. 2020 [17]	Siemens Centaur	31.1 ± 3.9	Chinese	Hong Kong	No/TPO	Yes (ICPM°)	NR**	12–13	16–20	32–36
Yuen et al. 2020 [17]	Beckman DXI	31.1 ± 3.9	Chinese	Hong Kong	No/TPO	Yes (ICPM°)	NR**	12–13	16–20	32–36
Ekinci et al. 2013 [18]	Beckman DXI	31 ± 4.7	Not reported	Australia	No/TPO	Yes (ICPM°)	129, 84, 71, 70***	9–13	22–26	35–39
Yuen et al. 2020 [17]	Abbott Architect	31.1 ± 3.9	Chinese	Hong Kong	No/TPO	Yes (ICPM°)	NR**	12–13	16–20	32–36
Ho et al. 2017 [19]	Abbott Architect	31.6 ± 4.6	Chinese	Singapore	NO/TPO, Anti-Tg	No	283	9–14	18–22	34–39
Ho et al. 2017 [19]	Abbott Architect	31.6 ± 4.7	Malay	Singapore	NO/TPO, Anti-Tg	No	143	9–14	18–22	34–39
Ho et al. 2017 [19]	Abbott Architect	31.6 ± 4.8	Indian	Singapore	NO/TPO, Anti-Tg	No	71	9–14	18–22	34–39
Ollero et al. 2019 [20]	Abbott Architect	33.4 ± 4 (29–37)	Caucasian	Spain	Yes/TPO, Anti-Tg	Yes (HPLC-EC°°°)	288, 252, 236****	9	15	36

Bold indicates data of the present study

NR = Not Reported

*Among 186 women

**600 pregnant women were recruited in their first trimester and were invited to have two more blood draws during subsequent antenatal visits, at least four weeks apart, and one final blood draw on admission for delivery. Blood samples collected in each woman were between 1 (n = 524) and 4 (n = 373). Number of subjects examined in the specified gestational weeks was not detailed

***Longitudinal data available in 47 women only, results mixed with other, cross-sectional samples

****Number of participants whose blood samples were available respectively in the 1st, 2nd and 3rd trimesters, among 291 women

°Inductively Coupled Plasma Mass Spectrometry

°°Sandell–Kolthoff reaction

°°°High performance liquid chromatography with electrochemical detection

**Table 5:** Trimester-specific reference intervals (2.5–97.5th percentile) for thyroid function in pregnancy, in the present and previous longitudinal studies. Post-partum (PP) reference intervals obtained from the same subjects are also reported, when available

Author, year publ (Refs.)	Method	TSH (mU/L)								FT4 (pmol/L)								FT3 (pmol/L)								
		1st		2nd		3rd		PP		1st		2nd		3rd		PP		1st		2nd		3rd			PP	
		LRL	UPR	LRL	UPR	LRL	UPR	LRL	UPR	LRL	UPR	LRL	UPR	LRL	UPR	LRL	UPR	LRL	UPR	LRL	UPR	LRL	UPR		LRL	UPR
**Present study**	**Roche Elecsys**	**0.34**	**3.81**	**0.68**	**4.07**	**0.63**	**4.00**	**0.56**	**3.42**	**11.40**	**17.40**	**9.98**	**15.25**	**9.49**	**15.1**	**11.9**	**19.3**	**3.88**	**6.05**	**3.61**	**5.38**	**3.59**	**5.37**		**4.2**	**6.5**
Sekhri et al. 2016 [13]	Roche Elecsys	0.09	6.65	0.51	6.66	0.91	4.86	–	–	9.81	18.53	8.52	19.43	7.39	18.28	–	–	3.1	6.35	2.39	5.12	2.57	5.68		–	–
Joosen et al. 2016 [14]	Roche Elecsys	0.11	3.39	0.25	3.38	0.51	3.85	0.53	2.92	11.70	20.00	9. .3	14.2	8.1	14.9	10.8	18.2	–	–	–	–	–	–		–	–
Ortega Carpio et al. 2018 [15]	Roche Elecsys	0.25	4.68	0.62	4.83	0.76	4.57	–	–	12.20	20.20	12	16.9	9	16.3	–	–	–	–	–	–	–	–		–	–
Kostecka-Matyja et al. 2017 [16]	Roche Elecsys	0.01	3.18	0.05	3.44	0.11	3.53	–	–	11.99	21.89	10.46	16.67	8.96	17.23	–	–	3.63	6.55	3.29	5.45	3.1	5.37		–	–
Yuen et al. 2020 [17]	Roche Elecsys	0.06	3.14	0.15	3.78	0.31	4.54	–	–	11.20	22.20	10.1	19.4	9	17	–	–	3.47	5.66	3.25	5.2	2.94	4.56		–	–
Yuen et al. 2020 [17]	Siemens Centaur	0.03	2.5	0.08	2.93	0.25	3.9	–	–	11.9	20.2	11.3	18.7	10.1	16	–	–	3.63	5.73	3.39	5.26	3	4.52		–	–
Yuen et al. 2020 [17]	Beckman DXI	0.11	2.71	0.19	3.25	0.3	3.87	–	–	8.20	14.40	7.4	12.6	6.7	11	–	–	4.04	6.14	3.85	5.75	3.57	5.18		–	–
Ekinci et al. 2013 [18]	Beckman DXI	0.03	3.05	0.42	3.36	0.34	2.83	0.25	2.55	5.90	15.50	4.9	11.3	4.5	11.20	4.5	14.4	3.90	5.7	3.7	5.2	3.5	5		3.8	6
Yuen et al. 2020 [17]	Abbott Architect	0.04	2.11	0.1	2.51	0.19	2.99	–	–	11.20	19.00	10.5	17.1	9.5	14.7	–	–	3.45	5.52	3.39	5.41	3.22	5.12		–	–
Ho et al. 2017 [19]	Abbott Architect	0.01	2.88	0.33	3.33	0.3	3.05	–	–	11.4	19.50	10.1	15.5	8.9	14.7	–	–	4.26	6.35	4.15	6.13	3.66	6.1		–	–
Ho et al. 2017 [19]	Abbott Architect	0.02	2.45	0.35	3.64	0.52	3.43	–	–	11.6	19.30	10	14.8	8.9	14.4	–	–	4.24	7.06	4.03	6.32	3.72	6.17		–	–
Ho et al. 2017 [19]	Abbott Architect	0.02	2.21	0.26	2.96	0.35	3.48	–	–	11.2	19.7	10.1	15.5	8.7	14.7	–	–	3.97	6.49	4.16	6.25	3.48	6.15		–	–
Ollero et al. 2019 [20]	Abbott Architect	0.13	4.16	0.31	3.73	0.58	4.36	–	–	10.94	15.96	10.55	15.44	8.62	13.64	–	–	–	–	–	–	–	–		–	–

Bold indicates data of the present study

## Discussion

The present study calculated the trimester-specific reference intervals for TSH, FT4 and FT3 in a carefully selected cohort of healthy Caucasian women living in a mild iodine deficiency area, in north-eastern Italy. To the best of our knowledge, this is the first longitudinal study in pregnancy and the post-partum that has assessed the reference intervals not only of TSH and FT4 but also of FT3, carried out with the Roche platforms.

The enormous amount of work and resources needed for a longitudinal design underpins the scarce number of prospective studies published. Smaller intra-individual variation of TFP during pregnancy has been reported by several authors [21–26] who advocated the development of longitudinal TFP reference intervals, although, a few studies [27, 28] did not report significant differences when comparing the self-sequential longitudinal and cross-sectional reference interval of thyroid function tests in pregnancy.

We found that there is a modest, but significant increase in TSH concentration in the second trimester of gestation, which remains stable thereafter in the third trimester, the median values being respectively 7.0% and 6.6% higher than in the late first trimester. Conversely, there was a clearer progressive reduction during pregnancy of FT4 and FT3 concentrations, the median values being 12.1% and 11.6% lower in the second trimester, and 14.4% and 13.2% lower in the third trimester, as compared with the first trimester, respectively. However, serum TSH, FT4 and FT3 concentrations were similar in the late first trimester and after the end of pregnancy.

Thyroid dysfunction is a frequent finding during pregnancy, which may have relevant medical implications [1, 3, 7]. It is noteworthy that even small variations in the thyroid function may be associated with adverse effects on several important pregnancy outcomes, including low birthweight and miscarriage risk. However, assessment of this condition requires the availability of appropriate reference intervals for thyroid function tests.

In most cases, TSH is considered the most important parameter in the assessment of thyroid function. However, the reference intervals for TH may also be clinically relevant for distinguishing an isolated thyroxine deficiency, a phenomenon possibly associated with potential unfavourable outcomes of pregnancy, and especially for properly diagnosing and managing any conditions of glandular hyperfunction. Indeed, the latter diagnosis may be challenging, due to the interfering effects of hCG, in the initial phase of gestation, and to the changes in FT4 and FT3 reference intervals during pregnancy. It is important to underline that pharmacological treatment of thyrotoxicosis is recommended in pregnant women when TH are increased, but not when there is only a suppressed serum TSH [8].

It should be emphasized that the differences in reference intervals of thyroid function parameters can be linked both to the methods used and to population-specific factors. It is therefore important to define reference intervals that are at least population and assay (analyzer and reagent) specific. This information is urgently needed from both a theoretical and practical point of view. While guidelines recommend the assessment of TFP in pregnancy using trimester- and instrumentation/reagents-specific reference intervals, this occurs very rarely in clinical practice, where clinical laboratories and clinicians usually adopt the intervals suggested by the manufacturers.

Medici et al. [29] and McNeil et al. [30] have highlighted in 2015 the heterogeneity of available studies in terms of methods, populations, iodine sufficiency status and gestational weeks of examined subjects. Interestingly, the first trimester TSH upper reference limit (URL) of the studies reviewed by McNeil et al. [30] fell into two groups: the mean of the values reported by the authors using Abbott, Beckman and Immulite-Siemens assays were around 3.0 mU/L (respectively 3.00, 3.12 and 3.09 mU/L), whereas the mean of the values reported by the authors using Centaur-Siemens and Roche were closer to 4.0 mU/L (respectively 3.55 and 4.00 mU/L).

The results reported in available longitudinal studies, summarized in Table 4, demonstrated different trends in TSH concentration: according to some studies, TSH is stable throughout pregnancy, according to others an increasing trend is recognizable. Conversely, a decreasing trend of FT4 (and FT3) concentration is more consistently reported.

In 2021 a systematic review of published studies on TSH and FT4 reference intervals in pregnancy obtained using Abbott, Beckman, Roche and Siemens assay methods, including 139,734 pregnant women, was conducted [31]. It is noteworthy that, in the first trimester, TSH upper limits obtained with the Abbott system ranged from 2.33 to 8.30 mU/L, those obtained with Siemens from 2.83 to 4.65 mU/L, whereas FT4 higher limits ranged from 13.2 to 18.7 pmol/L with the Beckman system, and from 16.7 to 26.5 pmol/L with the Siemens method. The TSH upper limit in the first trimester differed from non-pregnant concentrations, and could not be predicted or extrapolated from non-pregnant values.

A large variation in reference limits within the same assay, and the lower FT4 reference intervals using Beckman assay compared to the other assays were confirmed in the most recent systematic review and meta-analysis carried out by Osinga et al. [32] including 63,198 pregnant women. These authors also reported that about 15% of the studies included in their systematic review narrowed the 2.5th to 97.5th percentile reference intervals (mostly to the 5th to 95th percentile) and observed that in this case the upper limit of TSH decreased substantially (− 0.63, − 0.65, and − 0.73 mU/L in the first, second, and third trimester, respectively), with a considerable potential increase in the number of women diagnosed with subclinical hypothyroidism. However, in our opinion, the 2.5th to 97.5th percentile reference interval, which is adopted by most laboratory professionals, manufacturers and clinicians, and is recommended by current CLSI standards, should still be preferred to the 5th to 95th percentiles, to avoid risk of overdiagnosis and overtreatment of pregnant women, in the present absence of clear evidence that this change may lead to advantages in the clinical setting. Interestingly, the robust analysis of our data (Table 1) showed a minor effect on the upper limit of TSH (+ 0.14, − 0.05, and − 0.06 mU/L, in the first, second, and third trimester, respectively). These small differences may be due to the distinctive characteristics of our cohort, entirely constituted by carefully selected healthy women. Anyway, coupling robust and non-parametric methods could be an effective way to assess the effect of TSH right skewed distribution, without increasing dishomogeneity of the TFP reference interval studies.

In the present study TSH URL was around 4 mU/L throughout all pregnancy. As shown in Table 4, this finding is consistent with other studies carried out using Roche.

It is noteworthy that iodine intake has been poorly investigated in the past. Indeed, urinary iodine has not been measured in many studies summarized in Table 4 [14–16, 19] or, when measured, has not been assayed with reference technology [13, 20]. In studies reported in the review by Medici et al., it was sufficient only in two of the investigated cohorts and mild-moderately insufficient in all other studies [29]. In our cohort a mild iodine deficiency was found. It should be noted that the WHO-recommended thresholds of urinary iodine concentrations can only be used on a population basis, whereas these values are hardly applicable to assess the iodine status of the individual, due to the large variability of urinary iodine excretion. However, according to recent reports, reference limits are not significantly affected by iodine deficiency, when including mild to moderate iodine-insufficient participants (urinary iodine secretion 50–149 µg/L) [32].

In the present study, TSH lower reference limits in the first trimester were higher than those reported by other authors (Table 4). This may be accounted for by the relatively late phase of the first sampling in subjects enrolled in our study (14–16 gestational weeks), which differed from other studies. A reduction of serum TSH might be expected in very early pregnancy under the effect of high hCG levels. Consistently with this phenomenon, it should be noted that in our study the 90% CI of the low reference limit for first trimester TSH ranged between 0.07 and 0.550. The relatively late recruitment of pregnant women in our study could also potentially explain the differences between the TSH reference intervals calculated in our study and those reported in another longitudinal study carried out using Roche in a Caucasian population, in Poland [22], although the gestational weeks in which blood samples were obtained were not detailed in the latter study. However, it is noteworthy that, in this study, the lower reference limits of TSH remained unusually low throughout pregnancy (0.05 and 0.11 mU/L in the second and third trimester, respectively) despite the corresponding FT4 and FT3 reference limits being similar to our findings. Differences in TSH values between these studies are not easily explained. Unfortunately, this study did not report the reference intervals obtained in these women after the end of pregnancy.

Apart from TSH, changes during pregnancy in TH concentrations must be considered in order to avoid the potentially harmful misinterpretation of clinical findings. Indeed, in our study both FT4 and FT3 concentrations showed a progressive reduction during physiological pregnancy, the median values being about 11.5–12% lower in the second trimester, and 13–14% lower in the third trimester, as compared with values measured in the first trimester and in non-pregnant subjects.

The strengths of our study are the careful selection of subjects (healthy women with a physiological pregnancy and healthy newborns), the longitudinal collection of blood samples during the different trimesters of pregnancy in the same individuals, and the comparison of pregnancy data with hormone concentrations after the end of pregnancy, in a subgroup of these women. It should be underlined that studies investigating TFP reference interval in pregnancy have been rarely carried out longitudinally in carefully selected women, and only a few of them were methodologically accurate and complete. Notwithstanding the TSH not normal distribution of values, the reference limits obtained in the three trimesters using non-parametric analysis were substantially confirmed using robust method, scarcely affected by skewness.

A limitation of the study is the small size of the sample. However, the CLSI EP28-A3c document endorses for nonparametric method to collect samples from a number of qualified reference individuals sufficient to yield a minimum of 120 samples. A further limitation, as it is for any published studies in this field, is that the reported reference limits could be appropriate in laboratories that serve the Caucasian population by using Roche analyzers, but not in laboratories that serve a different population or the same population using analyzers of other manufacturers.

## Conclusions

In conclusion, this study illustrates the trimester-specific reference limits for TSH, FT4 and FT3 that should be used for the currently available Roche assay in Caucasian women, especially for those living in north-eastern Italy, who show mild iodine deficiency. Our results confirm that: (a) previously suggested fixed reference limits for TSH are unsuitable for many pregnant women; (b) the recent recommendation to indicate the used analyzer in the laboratory reports of tumour markers and hormones for increasing the stewardship capability of the laboratory professionals is particularly relevant in the reporting of the TFP in pregnancy [33].

### Supplementary Information

Below is the link to the electronic supplementary material.

Supplementary file1 (DOCX 24 KB)

Supplementary file2 (DOCX 18 KB)

Supplementary file3 (DOCX 18 KB)

## Data Availability

Some or all data sets generated during and/or analyzed during the present study are not publicly available but are available from the corresponding author on reasonable request.
