# Supplementary material for: Trimester-specific reference intervals for thyroid function parameters in pregnant Caucasian women using Roche platforms: a prospective study
Source: J Endocrinol Invest. 2023 Apr 24;46(12):2459–69. doi: 10.1007/s40618-023-02098-0 (PMC10632219; doi:10.1007/s40618-023-02098-0)
Supplement: Supplementary file 1 — Supplementary file1 (DOCX 24 KB) [file 40618_2023_2098_MOESM1_ESM.docx]

**Supplemental material 1**

**Papers assessing reference intervals of thyroid tests in pregnancy in samples not longitudinally collected.**

**A. Papers using Roche instrumentation**

1.1. Derakhshan A, Shu H, Broeren MAC, de Poortere RA, Wikström S, Peeters RP, et al. Reference Ranges and determinants of thyroid function during early pregnancy: the SELMA Study. J Clin Endocrinol Metab 2018;103:3548-56. doi:10.1210/jc.2018-00890.

1.2. Zhou Q, Zhang Y, Zhou J, Yang X, Huang Y, Li H, et al. Analysis of detection results of thyroid function-related indexes in pregnant women and establishment of the reference interval. Exp Ther Med 2019;17:1749-55. doi:10.3892/etm.2018.7135.

1.3. Khalil AB, Salih BT, Chinengo O, Bardies MRD, Turner A, Abdel Wareth LO. Trimester specific reference ranges for serum TSH and Free T4 among United Arab Emirates pregnant women. Pract Lab Med;12:e00098. doi:10.1016/j.plabm.2018.e00098.

1.4. Donovan LE, Metcalfe A, Chin A, Yamamoto JM, Virtanen H, Johnson JA, et al. A practical approach for the verification and determination of site- and trimester-specific reference intervals for thyroid function tests in pregnancy. Thyroid 2019; 29: 412-420. doi:10.1089/thy.2018.0439.

1.5. Gao X, Li Y, Li J, Liu A, Sun W, Teng W, et al. Gestational TSH and FT4 reference intervals in Chinese women: a systematic review and meta-analysis. Front Endocrinol (Lausanne) 2018;9:432. doi:10.3389/fendo. 2018.00432.

1.6. Morais NAOES, Assis ASA, Corcino CM, Saraiva DA, Berbara TMBL Ventura CDdD et al. Recent recommendations from ATA guidelines to define the upper reference range for serum TSH in the first trimester match reference ranges for pregnant women in Rio de Janeiro. Arch Endocrinol Metab 2018;62:386-91.

1.7. Liu J, Yu X, Xia M, Cai H, Cheng G, Wu L et al. Development of gestation-specific reference intervals for thyroid hormones in normal pregnant Northeast Chinese women: What is the rational division of gestation stages for establishing reference intervals for pregnancy women? Clin Biochem 2017;50:309-17.

1.8. Roche. Reference Intervals for children and adults. Cobas Thyroid Tests. Roche Diagnostics. Mannheim, Germany 2009.

1.9. Castillo C, Lustig N, Margozzini P, Gomez A, Rojas MP, Muzzo S, Mosso L. Thyroid-Stimulating Hormone reference ranges in the first trimester of pregnancy in an iodine-sufficient country. Endocrinol Metab 2018;33:466-72.

1.10. Andersen SL, Christensen PA, Knøsgaard L, Andersen S, Handberg A, Hansen AB, Vestergaard P. Classification of Thyroid Dysfunction in Pregnant Women Differs by Analytical Method and Type of Thyroid Function Test. J Clin Endocrinol Metab 2020;105(11):dgaa567. doi:10.1210/clinem/dgaa567.

1.11. Bunch DR, Firmender K, Harb R, El-Khoury JM. First- and Second-Trimester Reference Intervals for Thyroid Function Testing in a US Population. Am J Clin Pathol 2021;155:776-80.

1.12. Joosen AM, van der Linden IJ, de Jong-Aarts N, Hermus MA, Ermens AA, de Groot MJ. TSH and fT4 during pregnancy: an observational study and a review of the literature. Clin Chem Lab Med 2016;54:1239-46.

**B. Papers using Siemens instrumentation**

2.1. Zhang D, Cai K, Wang G, Xu S, Mao X, Zheng A, et al. Trimester-specific reference ranges for thyroid hormones in pregnant women. Medicine (Baltimore) 2019;98:e14245. doi:10.1097/MD.0000000000014245.

2.2. Kianpour M, Aminorroaya A, Amini M, Feizi A, Janghorbani M, Shokri S, et al. Reference intervals for thyroid hormones during the first trimester of gestation: a report from an area with a sufficient iodine level. Horm Metab Res 2019;51:165-71. doi:10.1055/a-0855-7128.

2.3. Dorizzi RM, Ozzola G, Sommella C, Catania F, Lelli F, Migali E, et al. An approach to establish reference intervals for thyrotropin in pregnancy using the ADVIA Centaur analyzer. Clin Lab 2010;56:417-25.

2.4. Ji C, Bu Y, Tian C, Fan L, Liu S, Liu Y, Sun D. Determination of reference intervals of ratios of concentrations of urinary iodine to creatinine and thyroid hormone concentrations in pregnant women consuming adequate iodine in Harbin, Heilongjiang Province. Biol Trace Elem Res. 2020;193:36-43.

2.5. Han L, Zheng W, Zhai Y, Xie X, Zhang J, Zhang S. Reference intervals of trimester-specific

thyroid stimulating hormone and free thyroxine in Chinese women established by experimental and statistical methods. J Clin Lab Anal 2018;32:e22344.

2.6. Friis Petersen J, Friis-Hansen LJ, Jensen AK, Nyboe Andersen A, Løkkegaard ECL [Early pregnancy reference intervals; 29 serum analytes from 4 to 12 weeks' gestation in naturally conceived and uncomplicated pregnancies resulting in live births.](https://pubmed.ncbi.nlm.nih.gov/31343977/) Clin Chem Lab Med 2019;57:1956-67.

2.7. Gilani M, Asif N, Akram A, Gilani M, Ijaz A, Malik SS. Determination of reference intervals of thyroid markers during pregnancy in urban area of district Rawalpindi Pakistan. J Pak Med Assoc 2018;68:1488-92.

2.8. Sheng Y, Huang D, Liu S, Guo X, Chen J, Shao Y, Zhang G, Wei L, Zeng X, Qiu X1. Reference intervals of thyroid hormones and correlation of BMI with thyroid function in healthy Zhuang ethnic pregnant women. BioMed Res Int 2018, 2018:2032413. doi:10.1155/2018/2032413.

2.9. Andersen SL, Andersen S, Carle´ A, Christensen PA, Handberg A, Karmisholt J, Knøsgaard L, Kristensen SR, Pedersen IB, Vestergaard P. Pregnancy week-specific reference ranges for thyrotropin and free thyroxine in the North Denmark Region Pregnancy Cohort. Thyroid 2019;29:430-8. doi:10.1089/thy.2018.0628.

2.10. Chen H-M, Kuo F-C, Chen C-C, Wu C-F, Sun C-W, Chen M-L, et al. New trimester specific reference intervals for clinical biochemical tests in Taiwanese pregnant women-cohort of

TMICS. PLoS ONE 2020;15:e0243761.

- 1. Canovi S, Vezzani S, Polese A, Frasoldati A, Schiatti C, Preda C, Corradini Zini M, Vitiello A, Foracchia M, Comitini G, Aguzzoli L, Fasano T, Vecchia L. Pregnancy-related reference intervals for serum thyrotropin based on real-life clinical data. Gynecol Endocrinol 2021;37:113-6.
  2. Gao X, Li Y, Li J, Liu A, Sun W, Teng W, Shan Z. Gestational TSH and FT4 reference intervals in Chinese women: a systematic review and meta-analysis. Front Endocrinol (Lausanne) 2018;9:432. doi:10.3389/fendo.2018.00432.
  3. Huang C, Wu Y, Chen L, Yuan Z, Yang S, Liu C. Establishment of assay method- and trimester-specific reference intervals for thyroid hormones during pregnancy in Chengdu, China. J Clin Lab Anal 2021;35:e23763.

**C. Papers using Beckman instrumentation**

3.1 Gao X, Li Y, Li J, Liu A, Sun W, Teng W, et al. Gestational TSH and FT4 reference intervals in Chinese women: a systematic review and meta-analysis. Front Endocrinol (Lausanne) 2018;9:432. doi:10.3389/fendo.2018.00432.

3.2 Liu J, Yu X, Xia M, Cai H, Cheng G, Wu L et al. Development of gestation-specific reference intervals for thyroid hormones in normal pregnant Northeast Chinese women: What is the rational division of gestation stages for establishing reference intervals for pregnancy women? Clin Biochem 2017;50:309-17.

3.3. Ren F, Zhou H, Chen M, Xiao X, Rui X. Comparative analysis of thyroid function parameters in pregnant women. Biomed Rep. 2017;7:455-9. doi:10.3892/br.2017.992.

3.4. Sun R, Xia J. The reference intervals of thyroid hormones for pregnant women in Zhejiang Province. Lab Med 2017;49:5-10.

3.5. Kim HJ, Cho YY, Kim SW, Kim TH, Jang HW, Lee S-Y, Choi S-Y, Roh C-R, Kim J-H, Chung JH, Oh S-y. Reference intervals of thyroid hormones during pregnancy in Korea, an iodine-replete area. Korean J Intern Med 2018;33:552-60.

3.6. Wang G, Zhang G. The diverse upper reference limits of Serum Thyroid-Stimulating Hormone on the same platform for pregnant women in China. Lab Med 2020;51:416-22.

**D. Papers using Abbott instrumentation**

4.1. Ly LD, Vuong NT, Chau MN, Phan HH, Pham QT, Pham TD, Le CH, Dang VQ. Reference intervals of thyroid function tests in first trimester Vietnamese pregnant women. Clin Lab 2020 Dec 1;66(12). doi:10.7754/Clin.Lab.2020.200415.

4.2. Akarsu S, Akbiyik F, Karaismailoglu E, Dikmen ZG. Gestation specific reference intervals for thyroid function tests in pregnancy. Clin Chem Lab Med 2016;54:1377-83.

4.3. Yang X, Meng Y, Zhang Y, Zhang C, Guo F, Yang S, et al. Thyroid function reference ranges during pregnancy in a large Chinese population and comparison with current guidelines. Chin Med J 2019;132:505-11.

4.4. Šálek T, Dhaifalah I, Langova D, Havalová J. Maternal thyroid-stimulating hormone reference ranges for first trimester screening from 11 to 14 weeks of gestation. J Clin Lab Anal 2018;32:e22405.
