# Supplementary material for: Trimester-specific reference intervals for thyroid function parameters in pregnant Caucasian women using Roche platforms: a prospective study
Source: J Endocrinol Invest. 2023 Apr 24;46(12):2459–69. doi: 10.1007/s40618-023-02098-0 (PMC10632219; doi:10.1007/s40618-023-02098-0)
Supplement: Supplementary file 2 — Supplementary file2 (DOCX 18 KB) [file 40618_2023_2098_MOESM2_ESM.docx]

**Supplemental material 2**

**Reference interval**

A reference interval (RI) for a parameter is the interval that includes the central 95% values of apparently healthy subjects. When both low and high values are clinically relevant 2.5% of healthy subjects are below and 2.5% of are above respectively lower and upper reference limit. A 90% confidence interval (CI) is calculated for both reference limits and will be more narrow with higher number of subjects in the study.

The RI can be estimated using 3 different methods: the Normal distribution method, a non-parametrical percentile method, and a "robust method".

**Normal distribution method**

In the Normal distribution method, the mean, variance, and standard deviation (SD) of the sample data are calculated. For a 2-sided RI, the reference limits are: lower = mean - 1.96 * SD and upper = mean + 1.96 * SD and the 90% confidence interval for each limit is given according to Bland.^1^ The normal distribution method requires that the data present a normal distribution, possibly after logarithmic or Box-Cox transformation, and does not require a minimum number of subjects, but a minimum sample size of 40 is recommended. ^2^

**Percentile method**

In the percentile method, the lower and upper reference limits of normality are given by the 2.5th and 97.5th percentiles for a double sided reference interval and the 5th percentile for a left sided reference interval, and 95th percentile for a right sided reference interval. According to the Clinical and Laboratory Standards standard EP28-A3c^3^ a minimal sample size of 120 subjects is required for calculating 90% CI in the percentile method defined using the method of Reed et al.^4^

**Robust method**

If sample size constraints of the simple nonparametric method prevent a laboratory from establishing reference intervals the CLSI working group recommends use of either bootstraps-based procedures or the robust methods “a compromise between the parametric and nonparametric methods since they do not require as many observations as the nonparametric procedure and do not require that the analytical values follow a gaussian distribution. It has the same form as the parametric but measure robust measures of location and spread instead of the mean and standard deviation”.

The robust method has been used as an alternative to the percentile method when sample size is less than 120 and when the underlying population is not assumed to follow a Gaussian distribution. ^3^ The computation involved has been detailed by Horn and Pesce. ^5^ The calculation of a reference interval using the robust method involves an iterative process, in which the initial central value is estimated by the median and the initial spread by the Median Absolute Deviation about the median (MAD). In the iterative process, actual observations are downweighted according to their distance from the central tendency of the sample. In each iteration, a quantity T^bi^, representing the updated estimate of central tendency, is calculated, until the change in consecutive iterative values is negligible. A 90% confidence interval is estimated using bootstrapping. ^6^

**References**

1. Bland M (2000) An introduction to medical statistics, 3rd ed. Oxford University Press, Oxford
2. Le Boedec K (2019) Reference interval estimation of small sample sizes: A methodologic comparison using a computer-simulation study. Vet Clin Pathol 2019;48 :335-346
3. Clinical and Laboratory Standards (CLSI) (2008) Defining, establishing and verifying reference intervals in the clinical laboratory; approved guideline-Third edition. CLSI document EP28-A3c. Wayne, Penn USA.
4. Reed AH, Henry RJ, Mason WB (1971) Influence of statistical method used on the resulting estimate of normal range. Clin Chem 17:275-284.
5. Horn PS, Pesce AJ, Copeland BE (1998) A robust approach to reference interval estimation and evaluation. Clin Chem 44: 622-631
6. Manual. Reference intervals. (2023) MedCalc version 20.218 https://www.medcalc.org/manual/referenceinterval.php Accessed 3 March 2023
