# Supplementary material for: Trimester-specific reference intervals for thyroid function parameters in pregnant Caucasian women using Roche platforms: a prospective study
Source: J Endocrinol Invest. 2023 Apr 24;46(12):2459–69. doi: 10.1007/s40618-023-02098-0 (PMC10632219; doi:10.1007/s40618-023-02098-0)
Supplement: Supplementary file 3 — Supplementary file3 (DOCX 18 KB) [file 40618_2023_2098_MOESM3_ESM.docx]

**Supplemental material 3**

Table A. Descriptive statistical analysis of TSH (mU/L) in each trimester of pregnancy and in the post-partum.

|  | First trimester | Second trimester | Third Trimester | Post-partum |
| --- | --- | --- | --- | --- |
| Sample size | 139 | 139 | 139 | 55 |
| Lowest value | 0,074 | 0.583 | 0.323 | 0.488 |
| Highest value | 4.810 | 4.690 | 4.370 | 3.450 |
| Arithmetic mean | 1.863 | 1.994 | 1.986 | 1.615 |
| 95% CI for Arithmetic mean | 1.714-2.022 | 1.854-2.135 | 1.845-2.127 | 1.427-1.804 |
| Median | 1.690 | 1.890 | 1.840 | 1.430 |
| 95% CI for the median | 1.519-1.985 | 1.645-2.095 | 1.710-2.025 | 1.313-1.726 |
| Variance | 0.839 | 0.702 | 0.706 | 0.485 |
| Standard deviation | 0.916 | 0.838 | 0.840 | 0.696 |
| Relative standard deviation | 0.49 | 0.42 | 0.42 | 0.431 |
| Standard error of the mean | 0.077 | 0.071 | 0.071 | 0.094 |
| Coefficient of Skewness | 0.581 (p=0.006) | 0.648 (p=0.003) | 0.639 (p=0.003) | 0.711 (p=0.031) |
| Coefficient of Kurtosis | 0.134 (p=0.620) | 0.200 (p=0.522) | 0.111 (p=0.660) | -0.082 (p=0.942) |
| Kolmogorov-Smirnov test for Normal distribution | Reject Normality (P=0.0103) | Reject Normality (P=0.018) | Reject Normality  (P=0.0331) | Reject Normality (P=0.0386) |

Table B. Descriptive statistical analysis of FT4 (pmol/L) in each trimester of pregnancy and in the post-partum

|  | First trimester | Second trimester | Third Trimester | Post-partum |
| --- | --- | --- | --- | --- |
| Sample size | 139 | 139 | 139 | 55 |
| Lowest value | 10.08 | 9.24 | 8 | 11.93 |
| Highest value | 19.05 | 16.9 | 16.25 | 19.45 |
| Arithmetic mean | 14.192 | 12.416 | 12.17 | 14.94 |
| 95% CI for Arithmetic mean | 13.935-14.449 | 12.191-12.642 | 11.936-12.416 | 14.46-15.437 |
| Median | 14.12 | 12.41 | 12.08 | 14.68 |
| 95% CI for the median | 13.8-14.376 | 12.078-12.651 | 11.819-12.626 | 14.175-15.464 |
| Variance | 2.348 | 1.842 | 2.047 | 3.261 |
| Standard deviation | 1.532 | 1.347 | 1.431 | 1.806 |
| Relative standard deviation | 0.108 | 0.108 | 0.117 | 0.12 |
| Standard error of the mean | 0.13 | 0.114 | 0.121 | 0.243 |
| Coefficient of Skewness | 0.277(p=0.173) | 0.350 (p=0.088) | 0.186 (p=0.355) | 0.504 (p=0.114) |
| Coefficient of Kurtosis | 0.076 (p=0.721) | 0.580 (p=0.172) | 0.133 (p=0.623) | -0.244 (p=0.864) |
| Kolmogorov-Smirnov test for Normal distribution | Accept Normality (P>0.1) | Accept Normality (P>0.1) | Accept Normality (P>0.1) | Accept Normality (P=0.086) |

Table C. Descriptive statistical analysis of FT3 (pmol/L) in each trimester of pregnancy and in the post-partum

|  | First trimester | Second trimester | Third Trimester | Post-partum |
| --- | --- | --- | --- | --- |
| Sample size | 139 | 139 | 139 | 55 |
| Lowest value | 3.46 | 3.32 | 3.36 | 3.9 |
| Highest value | 6.64 | 5.74 | 5.71 | 7.27 |
| Arithmetic mean | 4.993 | 4.446 | 4.388 | 5.18 |
| 95% CI for Arithmetic mean | 4.900-5.085 | 4.371-4.521 | 4.311-4.464 | 4.958-5.259 |
| Median | 4.990 | 4.41 | 4.33 | 5.13 |
| 95% CI for the median | 4.864-5.171 | 4.309-4.511 | 4.25-431 | 4.92-5.194 |
| Variance | 0.306 | 0.202 | 0.207 | 0.31 |
| Standard deviation | 0.553 | 0.449 | 0.455 | 0.557 |
| Relative standard deviation | 0.111 | 0.101 | 0.104 | 0.109 |
| Standard error of the mean | 0.047 | 0.038 | 0.038 | 0.075 |
| Coefficient of Skewness | -0.114 (p=0.570) | 0.262 (p=0.197) | 0.439 (p=0.034) | 0.951 (p=0.006) |
| Coefficient of Kurtosis | 0.115 (p=0.653) | 0.310 (p=0.383) | 0.212 (p=0.505) | 3.119 (p=0.004) |
| Kolmogorov-Smirnov test for Normal distribution | Accept Normality (P>0.1) | Accept Normality (P>0.1) | Accept Normality (P>0.1) | Reject Normality (P>0.1) |
